# From islands to infectomes: host-specific viral diversity among birds across remote islands

**DOI:** 10.1186/s12862-024-02277-4

**Published:** 2024-06-26

**Authors:** Rebecca M. Grimwood, Enzo M. R. Reyes, Jamie Cooper, Jemma Welch, Graeme Taylor, Troy Makan, Lauren Lim, Jérémy Dubrulle, Kate McInnes, Edward C. Holmes, Jemma L. Geoghegan

**Affiliations:** 1 Department of Microbiology and Immunology, University of Otago, Dunedin, 9016 New Zealand; 2 School of Medical Sciences, The University of Sydney, Sydney, NSW 2006 Australia; 3 Department of Conservation/Te Papa Atawhai, Nelson, New Zealand; 4 Institute of Environmental Science and Research, Wellington, 5018 New Zealand

**Keywords:** Birds, Chatham Islands, New Zealand, Infectome, Microbiome, Cross-species transmission, Virus

## Abstract

**Background:**

Accelerating biodiversity loss necessitates monitoring the potential pathogens of vulnerable species. With a third of New Zealand's avifauna considered at risk of extinction, a greater understanding of the factors that influence microbial transmission in this island ecosystem is needed. We used metatranscriptomics to determine the viruses, as well as other microbial organisms (i.e. the infectomes), of seven bird species, including the once critically endangered black robin (*Petroica traversi*), on two islands in the remote Chatham Islands archipelago, New Zealand.

**Results:**

We identified 19 likely novel avian viruses across nine viral families. Black robins harboured viruses from the *Flaviviridae*, *Herpesviridae*, and *Picornaviridae*, while introduced starlings (*Sturnus vulgaris*) and migratory seabirds (Procellariiformes) carried viruses from six additional viral families. Potential cross-species virus transmission of a novel passerivirus (family: *Picornaviridae*) between native (black robins and grey-backed storm petrels) and introduced (starlings) birds was also observed. Additionally, we identified bacterial genera, apicomplexan parasites, as well as a novel megrivirus linked to disease outbreaks in other native New Zealand birds. Notably, island effects were outweighed by host taxonomy as a significant driver of viral composition, even among sedentary birds.

**Conclusions:**

These findings underscore the value of surveillance of avian populations to identify and minimise escalating threats of disease emergence and spread in these island ecosystems. Importantly, they contribute to our understanding of the potential role of introduced and migratory birds in the transmission of microbes and associated diseases, which could impact vulnerable island-endemic species.

**Supplementary Information:**

The online version contains supplementary material available at 10.1186/s12862-024-02277-4.

## Introduction

In an era where an ever-increasing number of species teeter on the brink of extinction, monitoring the emergence of pathogens is an essential tool to inform conservation management. The avian world boasts an array of charismatic species, but few have stories rivalling that of the remarkable population recovery of the Chatham Island black robin (*Petroica traversi/*black robin/kākāruia/karure). First identified in 1872 as an insectivorous, forest-dwelling member of the Australasian robins (family: *Petroicidae*) endemic to the Chatham Islands (Rēkohu/Wharekauri), Aotearoa New Zealand [1], this small passerine holds a unique place in conservation history. By the early 1980s, their population had dwindled to just five individuals, bestowing them the title of “the rarest bird in the world” [2]. The survival of a solitary breeding pair, including the only successful breeding female, *Old Blue*, formed the foundation of ongoing conservation efforts to restore this species [3]. Today’s population has rebounded to over 300 birds, all descendants of *Old Blue*.

Despite their resurgence, the future of the black robin is still faced with uncertainty. New Zealand and its unique flora and fauna have evolved in near geographic isolation since splitting from the Gondwanan supercontinent some 85 million years ago (mya) [4]. The Chatham Islands, located around 800 km east of New Zealand’s South Island, have been an epicenter for the evolution of distinctive species since their emergence from the Chatham Rise roughly three mya [5], hosting at least 52 avian species, 18 of which occur nowhere else in the world [6]. It is here that, like most New Zealand endemics, black robins existed in geographic isolation devoid of natural predators for an extended period. Human colonisation of the Chatham Islands, followed by the arrival of introduced species, played a pivotal role in the black robin's decline [6, 7]. Now, due to their low genetic diversity, these robins are highly susceptible to the looming threats of infectious diseases such as highly pathogenic avian influenza viruses [8, 9].

Sedentary black robins co-exist and frequently interact with other avian species. Notably, seabirds (Procellariiformes) that breed in large numbers across the Chatham Islands serve as potential vectors for infectious agents [6]. While black robins remain isolated within their now limited home ranges on just two of five major forested islands in the archipelago, seabirds, on the other hand, act as biological bridges, potentially moving pathogens from one island to another through natal dispersal and roosting on different islands. In addition, common starlings (*Sturnus vulgaris*), an introduced passerine which has been implicated in the predation of black robin nests ([3], Reyes et al. In prep), pose another significant potential threat as vectors of disease transmission to black robins. Starlings forage on farmland on nearby Pitt Island and fly over to roost on the predator-free islands at night [3]. Determining the infectomes of these avian hosts, in conjunction with black robins, may provide a holistic view of potential transmission dynamics among these birds and deepen our understanding of future disease risks.

Avian viruses and wider infectomes remain vastly underexplored in New Zealand. While introduced pathogens such as avipoxvirus and *Plasmodium* spp. (avian malaria) are known to be circulating [10–12], only a few studies have integrated metagenomic approaches to identify all novel infectious agents in avian hosts [13–16]. Moreover, many of New Zealand's avian species grapple with limited genetic diversity, rendering them potentially susceptible to severe diseases and mortality from otherwise innocuous infections [17, 18].

Herein, we present a metatranscriptomic analysis of swab samples collected across seven bird species from Mangere and Rangatira Islands. By comparing the infectomes of sedentary black robins with those of other cohabiting and migratory species, we sought to gain a deeper understanding of the transmission dynamics and ecological factors influencing avian viromes and microbiomes in New Zealand. In particular, we aimed to determine whether birds with inter-island movements act as vectors, facilitating the transmission of microbes to and between species and islands. We emphasise the importance of understanding isolation and interconnectivity in these unique ecosystems, particularly in the context of cross-species transmission of pathogens to threatened species.

## Methods

### Ethics

This work has been approved by the New Zealand Department of Conservation as part of work carried out for species conservation purposes listed in annual business plans. Sample collection tasks that occur during these routine activities are authorised under Section 5(3) of the New Zealand Animal Welfare Act (1999). Standard Operating Procedures are followed for these tasks.

### Bird sampling and swab storage

*Petroica traversi* (black robin) and *Thinornis novaeseelandiae* (shore plover/tchuriwat’/tūturuatu) had oral and cloacal swabs taken during other work scheduled for management purposes, such as banding, by the Department of Conservation. Black robins were sampled on Mangere Island throughout the breeding season from December 2021 to February 2022 and on Rangatira Island during a post-breeding census from March to April 2022. Birds were primarily caught with pull-activated drop traps baited with mealworms and, where this was not possible due to a trap-shy individual, with a mist net. Shore plovers were sampled on Rangatira Island during a colour band maintenance trip in May 2022 and were caught with noose mats. Starlings (*Sturnus vulgaris*) and seabirds (Procellariiformes), including fairy prions (*Pachyptila turtur*), broad-billed prions (*Pachyptila vittata*), sooty shearwater (*Ardenna grisea*), and grey-backed storm petrels (*Garrodia nereis*), were caught opportunistically for sampling during the 2021-22 seasons field work on both islands with a minimum of five individuals per species collected on each island. Importantly, no obvious signs of disease were noted for any sampled birds. Oral and cloacal swabs were taken from individuals and stored in sterile tubes filled with 1 mL DNA/RNA shield (Zymo Research). In some cases, a fresh faecal sample was collected instead of obtaining a cloacal swab. Samples were sent to the Department of Microbiology and Immunology, University of Otago and stored at -80 °C until processing.

### Total RNA extraction and sequencing

Frozen oral and cloacal swabs from individual birds were defrosted and placed together in ZR BashingBead Lysis Tubes (0.1 and 0.5 mm) (Zymo Research) filled with 1 mL of fresh DNA/RNA shield (Zymo Research) using sterile forceps. Lysis tubes were homogenised for five minutes in a Mini-BeadBeater-24 disruptor (Biospec Products Inc.). Total RNA was then extracted following the ZymoBIOMICS MagBead RNA kit (Zymo Research). RNA concentrations were quantified using a NanoDrop Spectrophotometer (ThermoFisher). Equal quantities (10-15 μL) of RNA from individuals were pooled into 19 groups by species, sampling location (Mangere Island or Rangatira Island), and age group (adults or juveniles) (Table 1). To manage instances where there were a larger number of individuals per species and age group, these were divided into up to two pools per group for an average of around 10 individuals per pool. The Illumina Stranded Total RNA Prep with Ribo-Zero plus kit (Illumina) was used for library preparation of the pooled samples. Libraries were sequenced on the Illumina NovaSeq 6000 platform and 150 bp paired-end reads were generated.

**Table 1:** Overview of sample libraries

**Library**	**Species name**	**Common name**	**Location**	**Number of birds**	**Age**	**Collection date**
BM1	*Petroica traversi*	Black robin	Mangere	16	Adult	Dec 2021 – Feb 2022
BM2	*Petroica traversi*	Black robin	Mangere	11	Pullus	Dec 2021 – Feb 2022
OM1	*Pachyptila turtur*	Fairy prion	Mangere	5	Adult	Dec 2021 – Jan 2022
OM2	*Pachyptila turtur*	Fairy prion	Mangere	1	Chick	Dec 2021 – Jan 2022
OM3	*Sturnus vulgaris*	Common starling	Mangere	1	Adult	Dec 2021 – Jan 2022
OM4	*Sturnus vulgaris*	Common starling	Mangere	4	Chick	Dec 2021 – Jan 2022
OM5	*Ardenna grisea*	Sooty shearwater	Mangere	12	Adult	Dec 2021 – Jan 2022
OM6	*Ardenna grisea*	Sooty shearwater	Mangere	11	Adult	Dec 2021 – Jan 2022
OM7	*Pachyptila vittata*	Broad-billed prion	Mangere	3	Adult	Dec 2021 – Jan 2022
OM8	*Pachyptila vittata*	Broad-billed prion	Mangere	12	Chick	Dec 2021 – Jan 2022
OM9	*Garrodia nereis*	Grey-backed storm petrel	Mangere	6	Adult	Dec 2021 – Jan 2022
BR1	*Petroica traversi*	Black robin	Rangatira	16	Adult	Mar – Apr 2022
BR2	*Petroica traversi*	Black robin	Rangatira	16	Adult	Mar – Apr 2022
BR3	*Petroica traversi*	Black robin	Rangatira	16	Juvenile	Mar – Apr 2022
BR4	*Petroica traversi*	Black robin	Rangatira	17	Juvenile	Mar – Apr 2022
OR1	*Ardenna grisea*	Sooty shearwater	Rangatira	10	Adult	Apr – May 2022
OR2	*Pachyptila vittata*	Broad-billed prion	Rangatira	9	Adult	Apr – May 2022
OR3	*Pachyptila vittata*	Broad-billed prion	Rangatira	9	Adult	Apr – May 2022
SP1	*Thinornis novaeseelandiae*	Shore plover	Rangatira	16	Adult	May 2022

### Virus discovery and abundance estimation

Raw sequence reads were quality trimmed and then assembled *de novo* using Trinity (v2.11) [19] with the “trimmomatic” flag. Assembled contigs were screened against the NCBI non-redundant nucleotide (nt) and protein (nr) databases using BLASTn [20] and Diamond BLASTx (v2.02.2) [21] searches, respectively. A sequence similarity e-value cut-off of 1×10^-10^ was set for all searches to reduce false positive hits. Putative virus sequences were then manually screened with additional BLASTn and BLASTx searches using the online BLAST server [22]. Viruses were considered where top hits included other viruses with avian or vertebrate host assignments and excluded non-vertebrate-associated viruses or non-viral (e.g. bird genomic DNA) hits. For putative DNA viruses, nucleotide sequences of the viruses were screened against host reference genomes (where available) and the NCBI nt database to exclude endogenous viral elements (EVEs) or misassigned host sequences.

Viral transcript abundances were estimated using the “align and estimate” module within Trinity with the “prep reference” flag set. RNA-seq by Expectation-Maximization (RSEM) [23] was used as the abundance estimation method and Bowtie 2 [24] as the alignment method. Transcript abundances were standardised for inter-library comparisons by dividing RSEM counts by their respective sequencing library depths. To reduce incorrect assignment of viruses to libraries due to index hopping, shorter viral contigs sharing more than 99% nucleotide identity with a longer contig in another library or host species and a read count < 0.1% of the highest count for that virus across the other libraries were considered contamination due to index-hopping and excluded.

### Phylogenetic analysis and genome annotation

Evolutionary relationships of the viruses identified here were determined to infer host assignments. We assumed that viruses that clustered with other avian or vertebrate viruses in their respective phylogenies were likely to be infecting the birds sampled here (avian host viruses), while those associated with other host types (e.g. invertebrates, bacteria, fungi, plants, or environmental metagenomes) were unlikely to be replicating in their associated bird host and instead represented viruses from sources such as dietary, environmental, or sample processing contamination.

Phylogenies were estimated using protein sequences containing the highly conserved RNA-dependent RNA polymerase (RdRp) or DNA-dependent DNA polymerase (DdDp), or where viral genomes did not encode polymerases, such as those from the *Papillomaviridae*, major capsid proteins were used. Viruses that could be assigned down to at least order level based on their polymerase or capsid were aligned with their closest genetic relatives identified by BLAST as well as a representative range of viruses from their respective taxonomic orders or families collected from NCBI Taxonomy [25] using the L-INS-i algorithm in MAFFT (v7.450) [26]. Alignments were visualised in Geneious Prime (v2020.2.4) [27] and ambiguously aligned regions were trimmed using trimAL (v1.2) [28] with the “automated1” flag set. IQ-TREE (v.1.6.12) [29] with the LG amino acid substitution model and 1000 ultra-fast bootstrapping replicates [30] were used to estimate maximum likelihood trees for each taxon. The “alrt” flag was also added to perform 1000 bootstrap replicates for the SH-like approximate likelihood ratio test [31]. Phylogenetic trees were annotated in FigTree (v1.4.4) [32] and rooted at their midpoints.

Alignment and annotation of the Passerivirus GPS genome was done using an annotated genome of its closest relative, *Passerivirus A1* (NC_014411), as a reference and was performed in Geneious Prime using the L-INS-i algorithm in MAFFT.

### Virus nomenclature

Viruses were considered novel if they shared < 90% RdRp or DdDp protein identity with their closest known relative, or < 80% genome identity with previously described species. Novel viruses were provisionally named with either the common name or genus of the proposed host species. In the case of Passerivirus GPS, “GPS” denotes the initials of the genera of the three host species it was identified in (*Garrodia*, *Petroica*, and *Sturnus*).

### Non-viral microbe classification

Transcripts (i.e. active gene expression) of archaeal, bacterial, and eukaryotic microbes were determined using CCMetagen [33]. Reads were first mapped to the NCBI nucleotide database [34], excluding unclassified environmental microbes and cloning vectors, using KMA [35], ensuring a match to a reference was only counted if both paired-end reads mapped to the reference. The KMA output was then processed with CCMetagen to produce taxonomic classifications which were merged and filtered at the genus level.

CCMetagen outputs were imported into R and filtered to exclude viruses and microbial genera with abundances < 10 reads per million (RPM) across all libraries. A subset of the full filtered list, denoted “genera-of-interest” and comprising potentially pathogenic avian microbes or those that had been linked to disease in other native birds in New Zealand [36], was also screened. *De novo* assembled contigs were also manually screened for the genera-of-interest identified with CCMetagen with additional BLASTn searches and alignment to 16S and 18S ribosomal RNA (rRNA) nucleotide sequences of their closest hits to validate their presence in a sample.

### Ecological and microbial diversity analysis

The effects of host taxonomy (Passeriformes, Procellariiformes, or Charadriiformes) and sampling location (Mangere Island or Rangatira Island) on full family-level virome (exogenous viruses only) and non-viral genus-level microbiome compositions were assessed using non-metric multidimensional scaling (NMDS). Briefly, standardised virome and filtered non-viral microbiome abundances were normalised by library depth and distance matrices were created using the vdist function available in the vegan package [37] with Bray-Curtis dissimilarity as the distance measure. NMDS was then performed on the distance matrices using the metaMDS function from vegan. Permutational multivariate analysis of variance (PERMANOVA) with the adonis2 function from vegan was used to test for statistical significance of the effect of host taxonomy and location on the virome and microbiome distances. Ecological analysis of avian viromes and non-viral microbiomes was performed in R (v4.1.1) and graphs were plotted using ggplot2 [38].

Alpha diversity of full non-viral microbiomes for each library was measured as both richness and Shannon diversity, which considers both the number of genera (richness) and their abundances (evenness). Differences in microbiome alpha diversity between Passeriformes and Procellariiformes and between black robins from Mangere Island and Rangatira Island were assessed using Welch two-sample t-tests. Due to shore plovers only being represented by a single sample, they were excluded from comparisons of alpha diversity.

## Results

We used metatranscriptomics to reveal the combined oral and cloacal infectomes of seven bird species from Mangere and Rangatira Islands – both part of the Chatham Islands, New Zealand (Table 1) – to characterise their associated viruses and other microbes, and exposure to potential pathogens. We also investigated the factors that influence infectome composition and transmission dynamic patterns between hosts and islands.

### Metatranscriptomic sequencing

**Fig. 1:**
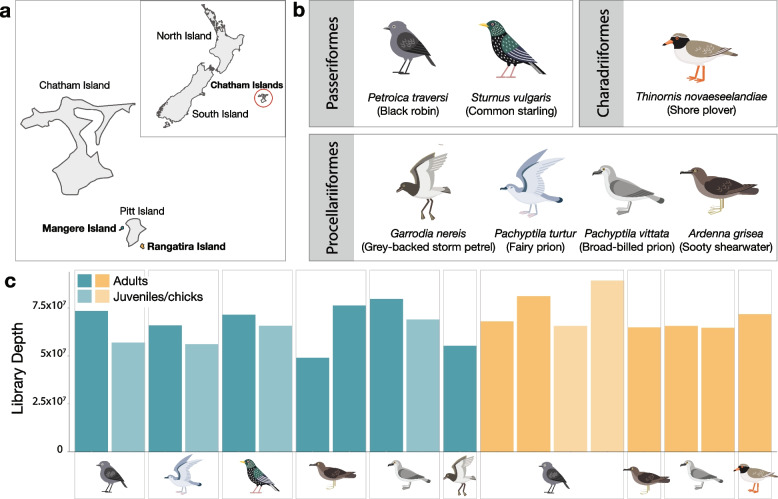
Overview of host species and sequencing. **a** The Chatham Islands, New Zealand. Mangere Island is bolded and highlighted in blue; Rangatira Island is bolded and highlighted in orange. **b** Seven avian species sampled, grouped by taxonomic order: Passeriformes, Charadriiformes, and Procellariiformes. **c** Sequence read depths of the 19 swab metatranscriptomic libraries. Host species are indicated by figures under the associated bars. Dark and light blue bars are adults and chicks from Mangere Island, respectively; dark and light orange bars are adults and chicks from Rangatira Island, respectively

High-throughput RNA sequencing of the 19 sequencing libraries (Table 1) generated approximately 49 to 89 million paired-end (150bp) reads per library (median = 66 million, Fig. 1). No quality issues were noted for any of the libraries. Raw reads were assembled *de novo*, generating 0.2 to 1.1 million contigs per library (median = 0.7 million) and viral reads accounted for around 0.003% to 12% of total sequence reads (median = 0.3%) following abundance estimations.

### Avian virome compositions and abundance

**Fig. 2:**
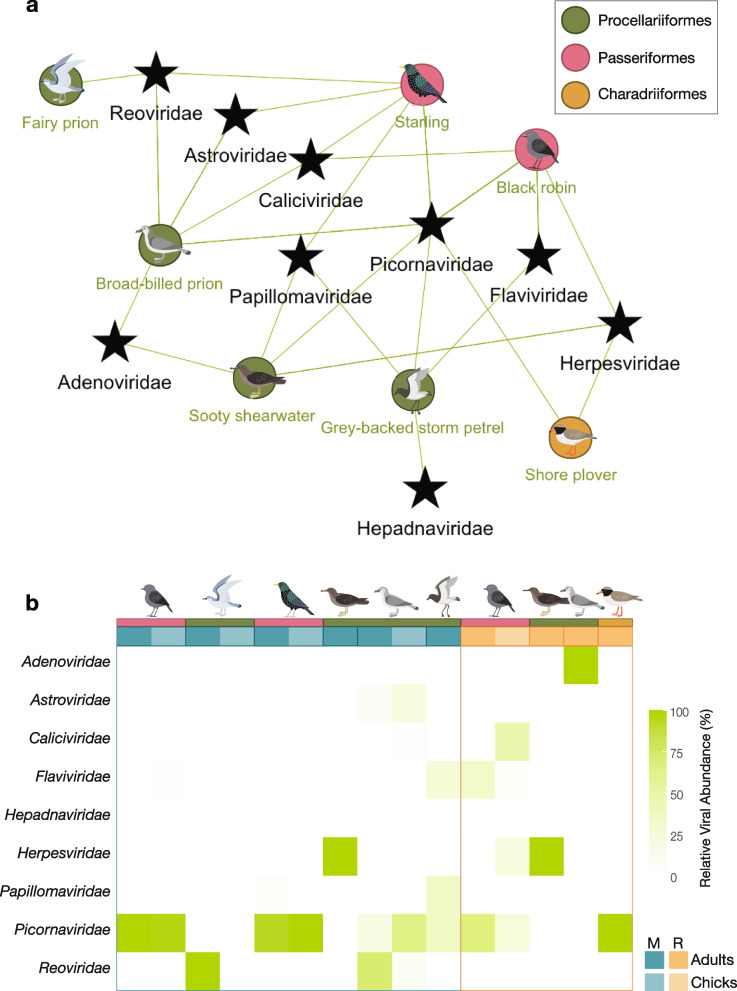
Diversity and relative abundances of avian viruses. **a** Bipartite network showing the connectivity of avian host species and their viromes. **b** Heatmap of relative abundances (%) of virus taxa by host species, location, and age group. Dark and light blue blocks indicate adult and chick viromes from Mangere Island (M), respectively; dark and light orange blocks indicate adult and chick viromes from Rangatira Island (R). Gradient from white to bright green indicates the relative abundance of viral taxa from 0 to 100% of the library’s total viral abundance

We identified sequences belonging to nine viral families across the seven hosts sampled (Fig. 2), with sequences from at least one avian virus identified per host species (range: one to five). Black robin viromes comprised vertebrate-specific viruses from the *Flaviviridae, Herpesviridae,* and the *Picornaviridae*, while starling and seabird viromes contained viruses from an additional six viral families. Only herpesvirus-like and picornavirus-like transcripts were identified in shore plovers from Rangatira Island (Fig. 2a and b). Viral transcripts across the *Picornaviridae* and *Herpesviridae* were among the most prevalent and abundant viral transcripts found across the Chatham Island birds sampled (Fig. 2b).

**Fig. 3:**
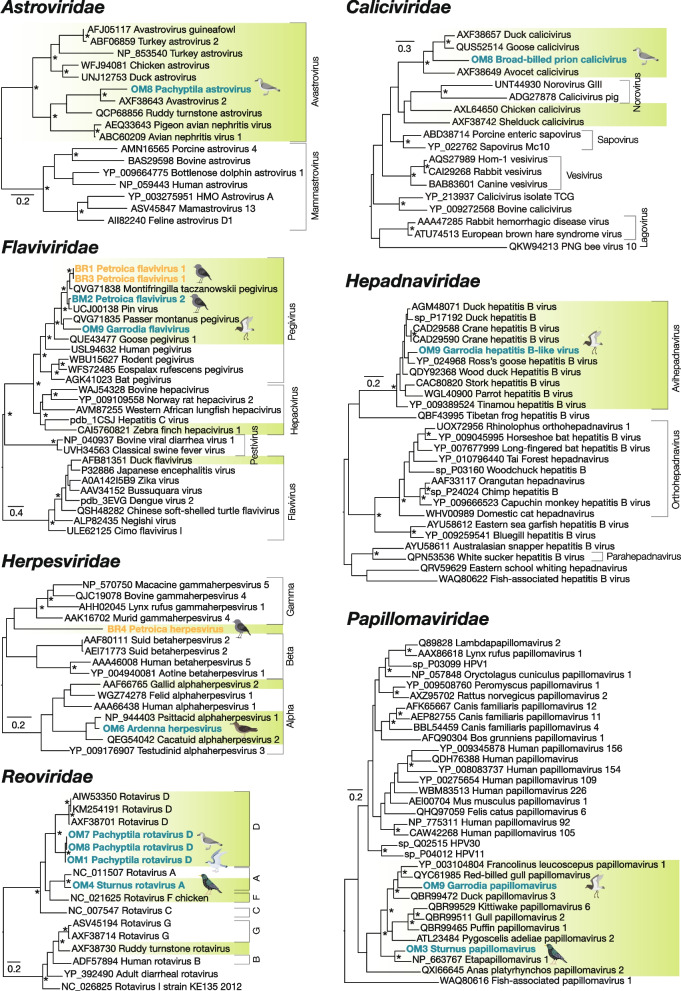
Maximum likelihood phylogenetic trees of avian viruses. Viruses identified in birds on Mangere Island are highlighted in blue and viruses identified on Rangatira Island are highlighted in orange. Green highlighted viruses indicate those with avian hosts. Substitutions per site are indicated by the keys on left-hand side of trees. All trees are rooted at their midpoint and nodes with ≥ 95 Ufbootstrap support values are denoted by an asterisk (*). Proposed host species of novel viruses are indicated by bird illustrations

**Fig. 4:**
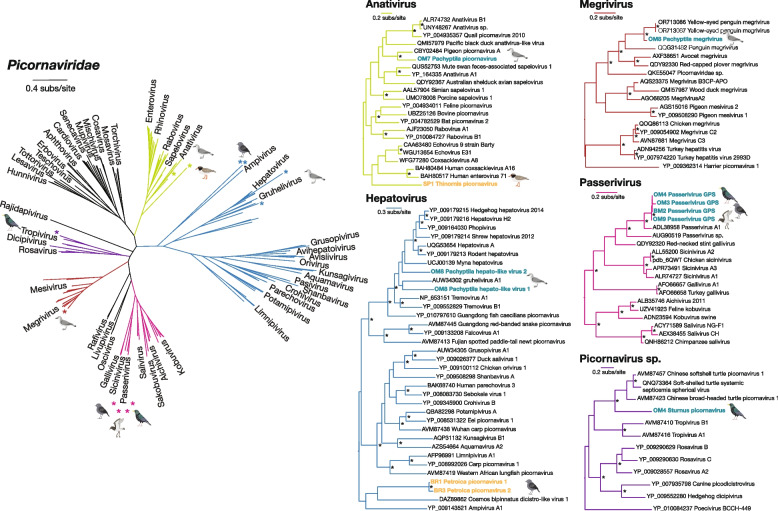
Maximum likelihood trees of avian viruses within the *Picornaviridae*. Overview of the *Picornaviridae* (left). Mid-point rooted trees of relevant *Picornaviridae* subfamilies and outgroups (right): *Anativirus* (green), *Megrivirus* (red), *Hepatovirus* (blue), *Passerivirus* (pink), and other *Picornavirus* species (purple). Nodes with ≥ 95 UFbootstrap support values are denoted by an asterisk (*). Viruses identified in birds on Mangere Island are highlighted in blue and viruses identified on Rangatira Island are highlighted in orange. Proposed host species are indicated by bird illustrations

In our analysis, we recovered amino acid sequences containing the viral capsid or polymerase, either the RdRp or DdDp, from 27 viruses sharing sequence similarity with known vertebrate host-associated viruses, allowing their phylogenetic relationships to be inferred and novelty to be determined. These sequences belonged to 19 distinct and likely novel avian virus species (Figs. 3 and 4 and Table 2). We also identified a virus each from the *Arenaviridae* and *Hantaviridae* families that were related to viruses from fish hosts in sooty shearwater from Mangere Island, and a hepevirus and picobirnavirus associated with black robins from Rangatira Island, all likely of environmental or dietary origin (Supplementary Figure 3). These four viruses were excluded from further discussion and analysis.

**Table 2:** Avian virus polymerase sequences^a^ described in this study

**Virus taxonomy**	**Host**	**Library**	**GenBank accession**	**Blastx top hit**	**Percentage identity (%)**	**Length (nt)**	**Novel?**
*Astroviridae*; Pachyptila astrovirus	*Pachyptila vittata*	OM8	OR645486	AXF38643.1 Avastrovirus 2	56	608	Yes
*Reoviridae*; Pachyptila rotavirus D	*Pachyptila turtur*	OM1	OR645487	APR73519.1 Rotavirus D	79.7	240	Yes^a^
*Reoviridae*; Sturnus rotavirus A	*Sturnus vulgaris*	OM4	OR645488	UNY48222.1 Rotavirus sp.	80.7	1887	Yes^a^
*Reoviridae*; Pachyptila rotavirus D	*Pachyptila vittata*	OM7	OR645489	AXF38701.1 Rotavirus D	67.1	2089	Yes^a^
*Caliciviridae;* Broad-billed prion calicivirus	*Pachyptila vittata*	OM8	OR645492	QUS52514.1 Goose calicivirus	53.6	303	Yes
*Herpesviridae*; Petroica herpesvirus	*Petroica traversi*	BR4	OR645493	AVQ93817.1 Human betaherpesvirus 6	41.3	272	Yes
*Herpesviridae*; Ardenna herpesvirus	*Ardenna grisea*	OM6	OR645494	NP_944402.1 Psittacid alphaherpesvirus 1	89.9	3252	Yes^a^
*Papillomaviridae*; Garrodia papillomavirus	*Garrodia nereis*	OM9	OR645495	NP_647590.1 Psittacus erithacus papillomavirus 1	60.7	873	Yes
*Papillomaviridae;* Sturnus papillomavirus	*Sturnus vulgaris*	OM3	OR900097	AYN76738.1 Etapapillomavirus 1	74.0	301	Yes
*Flaviviridae*; Petroica flavivirus 1	*Petroica traversi*	BR1	OR645496	QUE43478.1 Goose pegivirus	82.9	226	Yes
*Flaviviridae*; Petroica flavivirus 2	*Petroica traversi*	BM2	OR645498	UCJ00138.1 Pin virus	90	214	No^a^
*Flaviviridae*; Garrodia flavivirus	*Garrodia nereis*	OM9	OR645499	UCJ00138.1 Pin virus	52.4	920	Yes
*Picornaviridae*; Passerivirus GPS	*Sturnus vulgaris*	OM4	OR645505	YP_003853285.1 Passerivirus A1	80.7	7494	Yes
*Picornaviridae*; Petroica picornavirus 1	*Petroica traversi*	BR1	OR645502	UCJ00139.1 Myna hepatovirus	34.5	711	Yes
*Picornaviridae*; Petroica picornavirus 2	*Petroica traversi*	BR3	OR645503	UCJ00139.1 Myna hepatovirus	33.7	933	Yes
*Picornaviridae*; Sturnus picornavirus	*Sturnus vulgaris*	OM4	OR645506	QNQ73364.1 Soft-shelled turtle systemic septicemia spherical virus	49.4	286	Yes
*Picornaviridae*; Pachyptila picornavirus	*Pachyptila vittata*	OM7	OR645507	CBY02484.1 Pigeon picornavirus A	61.3	356	Yes
*Picornaviridae;* Pachyptila hepato-like virus 1	*Pachyptila vittata*	OM8	OR645508	ART66868.1 Hepatovirus A	44.8	846	Yes
*Picornaviridae;* Pachyptila hepato-like virus 2	*Pachyptila vittata*	OM8	OR645509	YP_009164030.1 Phopivirus	48	643	Yes
*Picornaviridae;* Pachyptila megrivirus	*Pachyptila vittata*	OM8	OR645510	QDY92330.1 Red-capped plover megrivirus	64.1	577	Yes
*Picornaviridae;* Thinornis picornavirus	*Thinornis novaeseelandiae*	SP1	OR900098	YP_164335.1 anativirus A1	38.6	795	
*Hepadnaviridae*; Garrodia hepatitis B-like virus	*Garrodia nereis*	OM9	OR645514	AJE59611.1 Duck hepatitis B virus	85.4	537	Yes

^a^If the virus was found in multiple libraries or species, the library with the longest sequence is detailed here. For a full breakdown of identified viruses see Additional file 6

### Evolutionary relationships of avian DNA viruses

Five novel virus transcripts were found across three double-stranded DNA (dsDNA) virus families: the *Hepadnaviridae* (*n*=1), *Herpesviridae* (*n*=2), and *Papillomaviridae* (*n*=2) (Fig. 3).

A putative exogenous hepatitis B-like virus polymerase segment was found in grey-backed storm petrels from Mangere Island. The virus, named Garrodia hepatitis B-like virus, fell within the genus of avian hepatitis B viruses, *Avihepadnavirus*, in the *Hepadnaviridae*. Garrodia hepatitis B-like virus shared 85% amino acid identity with *Duck hepatitis B virus* (DHBV) (AJE59611.1) (Table 2) and accounted for 0.014% of the total viral reads in the petrel library. Hepatitis B viruses, such as DHBV, infect hepatocytes in the liver of various bird species and can lead to persistent, lifelong infections if infected congenitally [39].

A partial herpesvirus DdDp from black robins on Rangatira Island and a full-length DdDp from sooty shearwater from Mangere Island were also identified. The divergent virus infecting black robins, Petroica herpesvirus, shared only 41% amino acid identity with *Human betaherpesvirus 6* (AVQ93817.1) but was phylogenetically placed outside of the *Gammaherpesvirinae* subfamily. No other avian-associated gamma or betaherpesviruses have been identified to date [40] and the virus was screened against the *Petroica traversi* genome assembly (GCA_025920805.1) to exclude it as an EVE. In contrast, the Ardenna herpesvirus DdDp in sooty shearwater shared almost 90% amino acid identity with that from *Psittacid alphaherpesvirus 1* (NP_944402.1) and fell within the *Alphaherpesvirinae* subfamily with other avian-associated herpesviruses. A full major capsid protein was also identified from the Ardenna herpesvirus (see Additional file 6). *Psittacid alphaherpesvirus 1* is the causative agent of Pacheco’s disease, a potentially lethal respiratory disease of parrots [41]. The divergence of the capsid and polymerase proteins from those of *Psittacid alphaherpesvirus 1*, in addition to the novel seabird host, suggests this new virus to be a distinct species. The black robin and sooty shearwater herpesviruses made up 0.017% and 62% of the viral abundances for the libraries they were identified from, respectively.

We identified a complete L1 segment of a papillomavirus, denoted Garrodia papillomavirus, in the grey-backed storm petrel library and a partial L1 segment from a starling library, named Sturnus papillomavirus, both from Mangere Island. Papillomaviruses are small non-enveloped dsDNA viruses that can produce a variety of benign and malignant epithelial lesions in animal hosts [42], primarily mammalian and bird species [43]. Around 0.34% of the total viral reads in the petrel were attributed to the novel papillomavirus, and it fell into an avian host-associated viral clade, sharing 61% amino acid identity with *Psittacus erithacus papillomavirus 1* (NP_647590.1), cloned from a papilloma of a grey parrot (*Psittacus erithacus*) [44]. The starling papillomavirus, on the other hand, comprised 0.003% of the total viral reads in the starling library and shared 74% amino acid identity with *Etapapillomavirus 1* (AYN76738.1) associated with skin lesions in wild British finches [45].

### Evolutionary relationships of avian RNA viruses

Several RdRps from positive-sense single-stranded RNA (+ssRNA) viruses from the *Astroviridae* (*n*=1), *Caliciviridae* (*n*=1), and *Flaviviridae* (*n*=3) families were identified in black robins and seabirds.

Members of the *Astroviridae* from mammalian and avian hosts can be divided into two distinct genera: *Mammastrovirus* (mammalian) and *Avastrovirus* (avian). Pachyptilla astrovirus from broad-billed prions fell into the genus *Avastrovirus*, exhibiting 56% amino acid identity with *Avastrovirus 2* (AXF38643.1) and comprised 0.048% of the total viral reads in the prion library. Avastroviruses, like *Avastrovirus 2*, can lead to avian nephritis, stunted growth, renal damage, gout and, rarely, mortality in poultry [46]. A segment of the structural polyprotein from the novel broad-billed prion astrovirus was also recovered (see Additional file 6).

Calicivirus sequences comprised approximately 0.01% of the viral abundance in a Mangere Island broad-billed prion library. We detected sequences of an RdRp sharing around 49% to 54% amino acid identity with avian-associated calicivirus species such as *Duck calicivirus* (AXF38657.1) and *Goose calicivirus* (QUS52514.1)*,* respectively, in the prions. Caliciviruses are not well described in wild birds [47] but can cause gastroenteritis or systematic disease in avian hosts [48].

Three pegi-like viruses (genus: *Pegivirus*) from the *Flavivirdae* were found in black robins from both islands and grey-backed storm petrel from Mangere Island, making up around 0.003 to 0.17% of the birds’ total viral abundances. The Rangatira black robin pegiviruses shared 63% to 89% identity with *Goose pegivirus* (QUE43478.), while the grey-backed storm petrel and Mangere black robin pegiviruses shared 53% to 90% amino acid identity with *Pin virus* from *Acridotheres tristis* (UCJ00138). Some flaviviruses, such as *goose pegivirus*, show lymphotropic pathogenicity and high rates of co-infection with astroviruses, parvoviruses, and circoviruses [49]. Other pegiviruses, such as the *Montifringilla taczanowskii pegivirus*, have been found in the respiratory tracts of passerines [50].

Rotaviruses, a group of double-stranded RNA (dsRNA) viruses from the *Reoviridae*, were identified in three species. We uncovered structural and non-structural sequences from Rotavirus D in broad-billed and fairy prions, as well as Rotavirus A in starlings, with polymerase-containing non-structural segments sharing 67-81% amino acid identity with other members of the genus *Rotavirus* (Fig. 3). Rotavirus D can cause enteric infections and stunted growth in birds and to date has only been detected in non-human animal hosts [51]. Rotavirus A is also a cause of major gastrointestinal disease in young birds and some genotypes may be able to infect mammalian hosts, including humans [52]. The rotavirus D viruses made up 0.021 to 0.18% of total viral reads for their respective libraries and fell above the threshold for exclusion due to index-hopping and the rotavirus A in starling made up 0.02% of viral reads in its library.

### The *Picornavirdae* and cross-species transmission of a novel *Passerivirus*

We identified nine likely novel virus species in the *Picornaviridae* across the subfamilies *Ensavirinae*, *Heptrevirinae* (hepatoviruses), and *Kodimesavirinae* (Fig. 4). Four of these were represented by novel virus transcripts related to bird-associated anativiruses, megriviruses and hepatoviruses found in broad-billed prions from Mangere Island. Of particular interest was Pachyptila megrivirus, which shared ~85% amino acid identity with a megrivirus recently discovered in diseased hoiho (yellow-eyed penguin) chicks from the Otago region of New Zealand (OR713086-95) [53] and made up 0.05% of total viral reads in its respective library.

Another notable observation was that of a novel passeri-like virus, Passerivirus GPS, in both passerines – black robins (endemic) and starlings (introduced) – and grey-backed storm petrels from Mangere Island. Passeriviruses have been linked to deaths in wild passerines and gastroenteric outbreaks in home-reared finches [54, 55]. Passerivirus GPS was highly abundant in a sample of four starling chicks (Fig. 2), accounting for around 88% of the total virus reads in the chicks. The virus was less abundant in the larger pools of black robin chicks and grey-backed storm petrel adults, comprising between 0.13 and 0.29% of the total viral reads for the libraries respectively. Moreover, a likely full genome was recovered from starling, including the full-length polyprotein (Supplementary Figure 1). The genome was aligned with its closest relative, *Passervirus A1* (NC_014411), from finches in Hungary for comparison. The two viruses shared approximately 78% genome (nt) identity and around 82% amino acid identity across the translated polyproteins, supporting Passerivirus GPS to be a novel species within the genus *Passerivirus*. Furthermore, the polyprotein of Passerivirus GPS (7,296 nt) was nine nucleotides longer than *Passerivirus A1* (7,287 nt), explained by four amino acid insertions in the L, VPO, VP1, and 2B peptides, and a deletion in the 3A peptide compared to its reference. Note that while the complete 3’ untranslated region (UTR) of the novel virus appeared to be recovered, including the poly-A tail, the 5’ UTR may be incomplete due to poor alignment and conservation of this region compared to its closest relative. The recovered viral segments in the two non-starling species shared 97.1 to 97.7% nucleotide identity with the starling polyprotein, suggesting recent cross-species transmission among these hosts. However, the petrel and robin segments did not overlap and therefore could not be compared directly.

### Presence and diversity of non-viral microbes

**Fig. 5:**
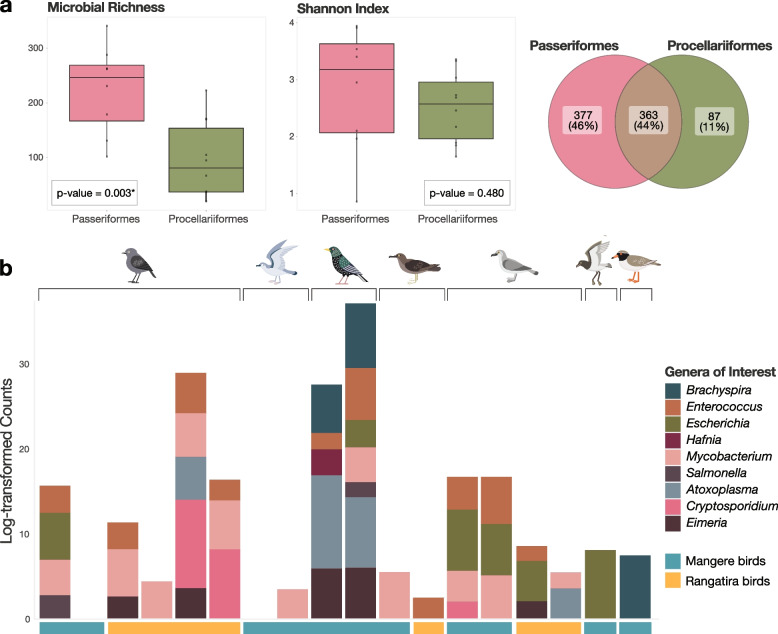
Non-viral microbe diversity and genera of interest. **a** Comparison of full genus-level microbiome microbial richness (left, t-test *p*-value 0.003), Shannon indices (middle, t-test *p*-value 0.480), and Venn diagram of unique genera (right) of Passeriformes (pink) and Procellariiformes (green). **b** Log-transformed read counts of nine microbial genera of interest, grouped by host species. Samples from Mangere Island are highlighted by blue boxes and those from Rangatira Island in orange boxes

Non-viral microbes present in the seven avian species, including archaea, bacteria, and eukaryotes, were characterised to assess overall microbial diversity and screen for significant avian pathogens (Fig. 5). Our analysis considered differences in full microbiome diversity and richness of the two most widely sampled groups (passerines and seabirds). Of the two groups, Passeriformes had significantly higher microbial richness (*p* = 0.003) than Procellariiformes, including 377 microbial genera unique to Passeriformes and only 87 unique to Procellariiformes (Fig. 5a). However, there was no statistically significant difference in Shannon diversity between the two groups.

Specific bacterial and eukaryotic genera were of particular interest as they have been associated with disease outbreaks and mortalities in other avian species in New Zealand due to environmental contamination, or because they contain notable avian pathogens [36, 56]. We identified the presence of microbes from nine of these genera of interest across the Chatham Island species (Fig. 5b). Both starling chicks and adults carried the largest number (eight out of nine) of these genera, while other species, such as the fairy prion, shore plover, and grey-backed storm petrels had none or only one of these genera present in their microbiomes. Passerines carried an average of 3.5 of these genera per group compared to 1.6 for Procellariformes groups, consistent with the patterns we observed in the diversity of full microbiomes. *Enterococcus*, *Escherichia,* and *Mycobacterium* were the most widespread of the genera across the birds sampled. Of further note was the high abundance (152 to 58,178 RPM) of parasites from the phylum *Apicomplexa* (including RNA sequencing reads from the genera *Eimeria*, *Cryptosporidium* and *Atoxoplasma*) in samples from both species in the Passeriformes (black robins and starlings) (Fig. 5b). Importantly, we found no evidence for the presence of significant avian pathogens such as *Plasmodium* spp., *Mycobacterium avium*, or *Erysipelothrix rhusiopathiae*. It should also be acknowledged that DNA sequencing could be used to complement the result of RNA analysis to provide a more comprehensive understanding of cellular microbiomes, particularly to confirm the presence of particular microbial species and strains identified in these birds.

### Effect of host taxonomy and location on microbiome composition

**Fig. 6:**
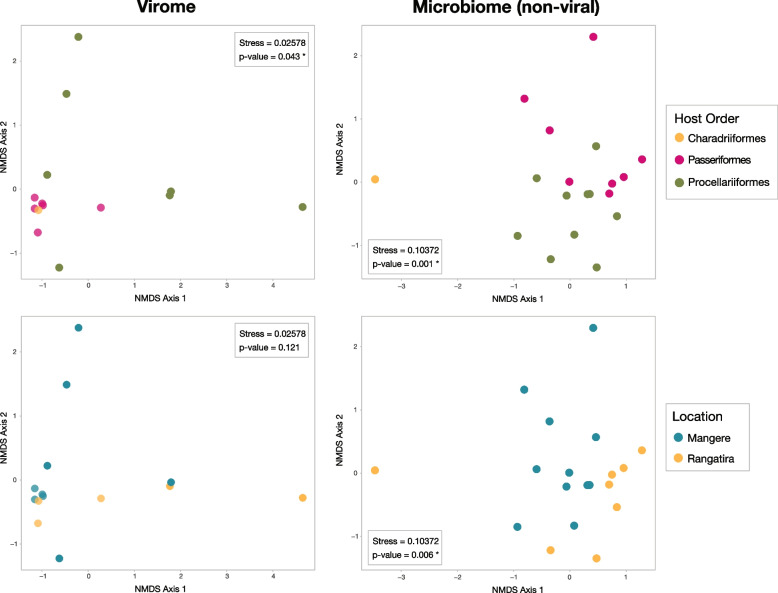
Non-metric multidimensional scaling plots of factors influencing avian microbial compositions. Avian-associated family-level virome compositions (left) and non-viral genus-level microbiome compositions (right) were plotted according to Bray-Curtis distances and coloured by host order (top) and sampling location (bottom). A random jitter of 0.1 was added to the x and y virome point values for pictorial clarity. Family-level virome composition was significantly influenced by host specificity (PERMANOVA *p*-value = 0.043), while non-viral microbiome compositions were significantly influenced by both host taxonomy and sampling location (PERMANOVA *p*-values = 0.001 and 0.006, respectively)

To examine potential differences in both avian family-level viromes and full genus-level non-viral microbiomes based on host taxonomy and sampling location, we conducted a multivariate analysis using host order and location (island) as key factors (Fig. 6). Bray-Curtis distances were used as the measure of sample microbial beta diversity. Host order was found to be significantly associated with both virome composition (*p* = 0.043) and non-viral (cellular) microbiome composition (*p* = 0.001) when controlling for sampling location. Sampling location was also found to have a significant impact on non-viral microbiomes (*p* = 0.006), with distinct clustering of samples by island, particularly samples from Rangatira Island, but not on virome compositions (*p* = 0.121). To further validate the effect of host factors on vertebrate virome composition, we repeated the analysis on the non-vertebrate viromes associated with the birds (i.e. viruses likely associated with their diet or environment and not directly infecting the birds themselves) as we assumed their presence would not be affected by avian biology and hence act as a negative control. Accordingly, we observed no significant effect of host order or location on non-vertebrate virome composition (Supplementary Figure 5).

## Discussion

Birds are hosts to a plethora of viruses and the migratory and dispersive lifecycles of some species make them important disease vectors, facilitating their transmission across large geographic distances [57, 58]. Birds are also a highly diverse group, represented by more than 10,000 species [59]. Despite this, studies of bird viruses and non-viral microbiomes are outnumbered ten-to-one by those of mammalian species [60]. With over a third of avian populations in Aotearoa New Zealand considered at risk or threatened [61], understanding the presence of microbes and the dynamics of their transmission is necessary to limit the potential impact of future disease outbreaks on these avifauna. We investigated the infectomes of a snapshot of the avian life on two isolated New Zealand islands to better understand the transmission and connectivity of microbes between species with differing behavioural ecology and taxonomy. Our findings detail the presence of known and novel microbes associated with the avifauna on the Chatham Islands, including factors that shape their infectomes. In addition, we present evidence for the transmission of a novel passerivirus between native and introduced sedentary and migratory birds.

We identified likely novel avian viruses in all seven hosts sampled, spanning nine viral families. Members of the *Flaviviridae*, *Herpesviridae,* and *Picornaviridae* were the dominant viruses in black robins. Other passerines in New Zealand have recently been shown to carry similar viral families with no evidence of transmission between native and introduced species [13]. However, another related robin species, the South Island robin (*Petroica australis*), has a large diversity of single-stranded DNA viruses which were absent in black robins sampled here [16]. We also uncovered additional viruses from the *Astroviridae*, *Caliciviridae*, *Hepadnaviridae*, *Papillomaviridae* and *Reoviridae* families in seabirds and starlings. While surveys of wild and invasive birds have also revealed similar viral families [49, 62], we have broadened the known host range and diversity of these virus families.

Some of the viruses we uncovered here are related to those that cause disease in other avian hosts. For example, the causative agent of Pacheco’s disease (family: *Herpesviridae*) has been reported in imported and captive parrots in New Zealand [63], and herein we identified a closely related alpha herpesvirus in sooty shearwater from Mangere Island. In contrast, the evolutionary history of the divergent herpesvirus in black robins from Rangatira Island is uncertain as no beta or gamma herpesviruses have been reported in Aves to date [40] and the possibility of contamination from mammalian species such as fur seals that haul out along coastal margins in the Chathams [64] close to black robin territories or from human interactions such as during routine population surveys over the years should not be ruled out. Similarly, rotaviruses are a common cause of enteric disease in animals, including humans [51, 52], and here, we detected these viruses in migratory seabirds and starlings.

The emergence and introduction of infectious diseases from migratory species threatens the maintenance of vulnerable populations. In this context, a major finding from this study was that we found a probable instance of cross-species transmission of a novel passerivirus (family: *Picornaviridae*), Passerivirus GPS, on Mangere Island among both passerine species (the black robin and introduced starling), as well as the native grey-backed storm petrel. Passeriviruses have previously only been reported in dead passerines, including wild birds from the *Turdidae* family and home-reared estrildid finches in association with gastroenteritis outbreaks [54, 55]. Hence, the presence of this virus in grey-backed storm petrels represents the first case of a passerivirus identified outside of passerine hosts. Here, we collected samples from seemingly healthy birds, although the high abundance of Passerivirus GPS in a library of mixed oral and cloacal swabs from four starling chicks is consistent with an enterically transmitted virus. It has been suggested that passeriviruses may be sustained in bird populations and only cause sporadic cases of disease [54], although prevalence data on asymptomatic birds is limited in wild populations. Increasingly, birds have been identified as vectors of viral pathogens without showing clinical signs [9, 58].

There are also gaps in the possible chain of transmission of Passerivirus GPS on the Chatham Islands that will provide important information on the natural ecology of these viruses. Transmission between starlings and black robins is likely due to excreta from starlings contaminating soil under the roosts where robins forage for invertebrates, interspecific interactions due to nest cavity competition, or potentially from the predation of robin nests by starlings [3], making a clear case for a viral cross-species transmission event between these taxonomically related birds. Grey-backed storm petrels, in contrast, have very different behavioural ecology. These small seabirds feed at sea and tend to nest in dense vegetation, while black robins and starlings dwell in forested areas [7]. While possible, the transmission of viruses between these species is less likely to be direct, instead involving other intermediate hosts not yet sampled. The high viral sequence similarity among these hosts also suggests relatively recent cross-species viral transmission. Sampling of the wider avifauna across the archipelago may help to identify missing links within these viral dynamics and confirm suspected hosts.

Among viruses that fell into the *Picornaviridae*, we identified viral transcripts related to anativiruses, hepatoviruses, and megriviruses, all of which have been previously documented in avian hosts [65] and are frequently pathogenic [66, 67]. Of particular note was the observation of a megrivirus in broad-billed prions from Mangere Island that was closely related to a megrivirus found in yellow-eyed penguins (*Megadyptes antipodes*) with diphtheritic disease from the Otago region, New Zealand [53]. Indeed, prions have relatively broad habitat distributions along southern New Zealand and the South Atlantic Ocean [68]. Hoiho and seabirds such as broad-billed prions breed on the same islands in the Foveaux Strait (McClellan R, Reid A, Pyatt T: Foveaux Strait seabirds: assessment of environmental effects for Project South, unpublished) which may have provided an environment for a historic cross-species viral transmission event to occur without the need for intermediary hosts. Therefore, the genetic proximity of the broad-billed prion megrivirus to other avian-associated viruses across wider New Zealand suggests that migratory birds play an important role in the transmission of these viruses and the interconnectedness of avian populations.

Few studies have directly compared the microbial diversity of seabirds and passerines, particularly where they co-exist in these isolated island ecosystems. Comparisons of the microbiomes of the most widely sampled host orders in this study - Passeriformes (passerines or perching birds) and Procellariiformes (seabirds) - revealed that Passeriformes had a larger number of distinct microbes than seabirds. Passerines have previously been shown to carry a number of unique microbial genera compared to non-passerine groups [69]. Through the detection of a subset of particular genera of interest, the presence of apicomplexan parasites in both passerine species was also highlighted. Parasites within this phylum, such as *Plasmodium relictum*, responsible for avian malaria, are relatively widespread in New Zealand birds, infecting around 34 species to date [70]. While no evidence of *P. relictum* was identified, evidence of other coccidian parasites, such as Atoxoplasma, were observed and are known to be very prevalent in other passerines, including other native robins [11, 14]. High parasitic loads may be harmful to their host, but they are rarely found in high enough abundance in wild populations to cause symptomatic disease. However, in the case of coinfections or young or immunocompromised inbred native birds, these parasites may pose a more urgent threat [71], making identifying and monitoring their prevalence in these vulnerable populations, despite seemingly healthy, a potentially important part of ongoing species management [72].

Microbiomes are influenced by a multitude of intrinsic and extrinsic factors [73]. The foraging, breeding and other behavioural ecology differed between taxonomic groups studied here. For example, seabirds and shorebirds (Charadriiformes) often roost on rocky coastlines or burrow under forest cover [6, 74] while passerines reside in forested areas and nest in tree cavities [6]. Furthermore, some passerines, like black robins, are entirely insectivorous [1, 2], while Charadriiformes are opportunistic feeders, sourcing small fish and invertebrates [75] and members of the order Procellariiformes hunt at sea for fish and krill [76]. Groups such as seabirds frequently travel large distances [7] and may come into contact with a larger number of different species than sedentary birds, such as black robins and shore plovers, presenting another key difference in behaviour that may impact differences in their microbiomes. Predictably, we found a significant host effect influencing both virome and non-viral microbiome beta diversity, while location also significantly influenced the diversity of non-viral microbiomes. Host taxonomy is frequently found to be the most significant impactor of virome composition [77], likely explained by viruses being obligatory intracellular parasites that depend on host factors such as specific cell receptor binding and replication machinery to infect and replicate within their host, and are therefore generally more host-specific. Contrastingly, non-viral microbiomes can be more generalised and are well documented as being more strongly influenced by non-host factors such as seasonality and geography [60, 78, 79], diet [80, 81], age [82], and sex [78, 83].

We note a relative lack of viruses in the Rangatira Island bird viromes, although this could represent disparities in processing, storage, and handling of swab samples between teams and islands [84], rather than a true absence of viruses. For example, during sampling in March to April when there are fewer daylight hours, fridges used for temporary storage may be turned off during days with poor weather conditions to conserve power. Some viruses, such as avipoxviruses, which are known to infect shore plovers and black robins on the Chatham Islands [12, 17, 63] were similarly not identified on either island, likely due to the types and body location of samples collected as skin biopsies or swabs taken directly from pox lesions are usually necessary to identify such viruses. Hence, body site bias would also influence what microbes may be identified, and therefore the findings presented here cannot rule out the presence or absence of other microbes that may have different tissue tropism.

## Conclusions

Through investigating the infectomes of the Chatham Island avian populations sampled we shed light on the role that birds play as reservoirs for a multitude of known and novel microbes and their potential to act as vectors for infectious agent transmission to threatened native species. Although we did not find evidence of viruses being transmitted between islands, the apparent cross-species transmission of a novel passerivirus between both native and introduced passerines and grey-backed storm petrels highlights the potential risks posed by introduced and migratory birds to the maintenance of vulnerable populations. Further sampling is needed to understand the prevalence and transmission dynamics of this virus, particularly given the migratory status of starlings and its apparent absence in the black robin population on Rangatira Island. Additionally, seabirds carried viruses related to other known or putative avian pathogens affecting native birds across wider New Zealand. Our comparative analysis of microbial diversity indicates that passerines carry a larger number of distinct microbial genera than seabirds and that host taxonomy outweighs location as a driver of virome composition. Since the Chatham Islands house a diverse avifauna, moving forward, future research should focus on expanding the species and numbers sampled and consider complementing RNA analysis of the cellular microbes with DNA sequencing to validate the trends observed here. Overall, this knowledge not only enhances our understanding of avian infectomes in New Zealand but also the role of introduced and migratory birds as possible vectors for future disease outbreaks that may impact threatened species, informing the broader field of conservation aimed at preserving the rich avian biodiversity of New Zealand [85].

### Supplementary Information

Additional file 1: Supplementary Figure 1. Polyprotein organisation of Passerivirus GPS from starlings. The recovered virus partial genome from a starling chick metatranscriptome (bottom) was annotated using its closest relative *Passerivirus A1* (top) as a guide. Four amino acid insertions within the L, VP0, VP2, and 2B peptides are denoted by pink right-pointing arrows and an amino acid deletion in the 3A peptide is denoted by an inverted pink arrow in comparison to the *Passerivirus A1* polyprotein. The translation of insertions and deletions are shown below. The height of pink bars (top) denotes nucleotide identity (%) between the two viruses.

Additional file 2: Supplementary Figure 2. Presence-absence plot of avian and avian-associated viral taxa by host species, location, and age group. Dark and light blue blocks indicate adult and chick viromes from Mangere Island (left), respectively; dark and light orange blocks indicate adult and chick viromes from Rangatira Island (right). Green blocks indicate the presence of avian viruses, while red blocks and viral taxa preceded by an asterisk (*) indicate avian-associated viruses, likely from dietary or environmental sources. Grey blocks indicate the presence of likely endogenous viral elements.

Additional file 3: Supplementary Figure 3. Maximum likelihood trees of avian-associated viruses. Viruses identified as being associated with birds on Mangere Island are highlighted in blue and viruses identified on Rangatira Island are highlighted in orange. Host species associated with the viruses are indicated by bird illustrations. Substitutions per site indicated by the key on left-hand side of trees. Nodes with ≥ 95 UFbootstrap support values are denoted by an asterisk (*).

Additional file 4: Supplementary Figure 4. Comparisons of non-viral microbial genera richness and diversity per host species and sampling location. Comparison of full genus-level microbiome microbial richness (left, t-test *p*-value 0.110) and Shannon indices (right, t-test *p*-value 0.117) of Mangere (blue) and Rangatira (orange) black robin (top). Venn diagrams of the unique microbial genera of black robin (left), broad-billed prions (middle), and sooty shearwater (right) based on their sampling location – Mangere (blue) or Rangatira (orange) (bottom).

Additional file 5: Supplementary Figure 5. Non-metric multidimensional scaling plots of non-vertebrate family-level virome compositions in Chatham birds. Non-vertebrate family-level virome compositions of sampling libraries (points) were plotted by Bray-Curtis distances and coloured by host order. Non-vertebrate family-level virome compositions were not significantly influenced by host taxonomy or location (PERMANOVA *p*-value = 0.08 and 0.65, respectively).

Additional file 6: Extended summary of microbes and virus segments identified. An excel spreadsheet containing extended information on the viruses and microbes identified in this study, including polymerase and non-polymerase segments and presence/absence of microbes in each library. 

## Data Availability

Raw sequence reads are available on the NCBI Short Read Archive (SRA) under the BioProject accession PRJNA1021626 while viral sequences identified are available under GenBank accession numbers OR645486 to OR645543 and OR900097 to OR900098. Please see GitHub for detailed R code and accompanying input data used to generate the results described in this study.
