# Supplementary figures and images for: From islands to infectomes: host-specific viral diversity among birds across remote islands
Source: BMC Ecol Evol. 2024 Jun 26;24:84. doi: 10.1186/s12862-024-02277-4 (PMC11209962; doi:10.1186/s12862-024-02277-4)

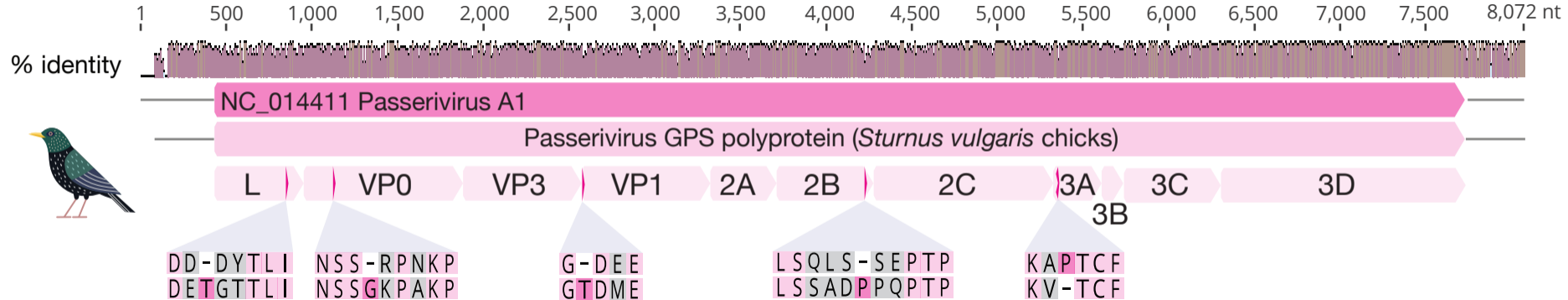

Supplement: Supplementary file 1 — Additional file 1: Supplementary Figure 1. Polyprotein organisation of Passerivirus GPS from starlings. The recovered virus partial genome from a starling chick metatranscriptome (bottom) was annotated using its closest relative Passerivirus A1 (top) as a guide. Four amino acid insertions within the L, VP0, VP2, and 2B peptides are denoted by pink right-pointing arrows and an amino acid deletion in the 3A peptide is denoted by an inverted pink arrow in comparison to the Passerivirus A1 polyprotein. The translation of insertions and deletions are shown below. The height of pink bars (top) denotes nucleotide identity (%) between the two viruses. [file 12862_2024_2277_MOESM1_ESM.pdf]

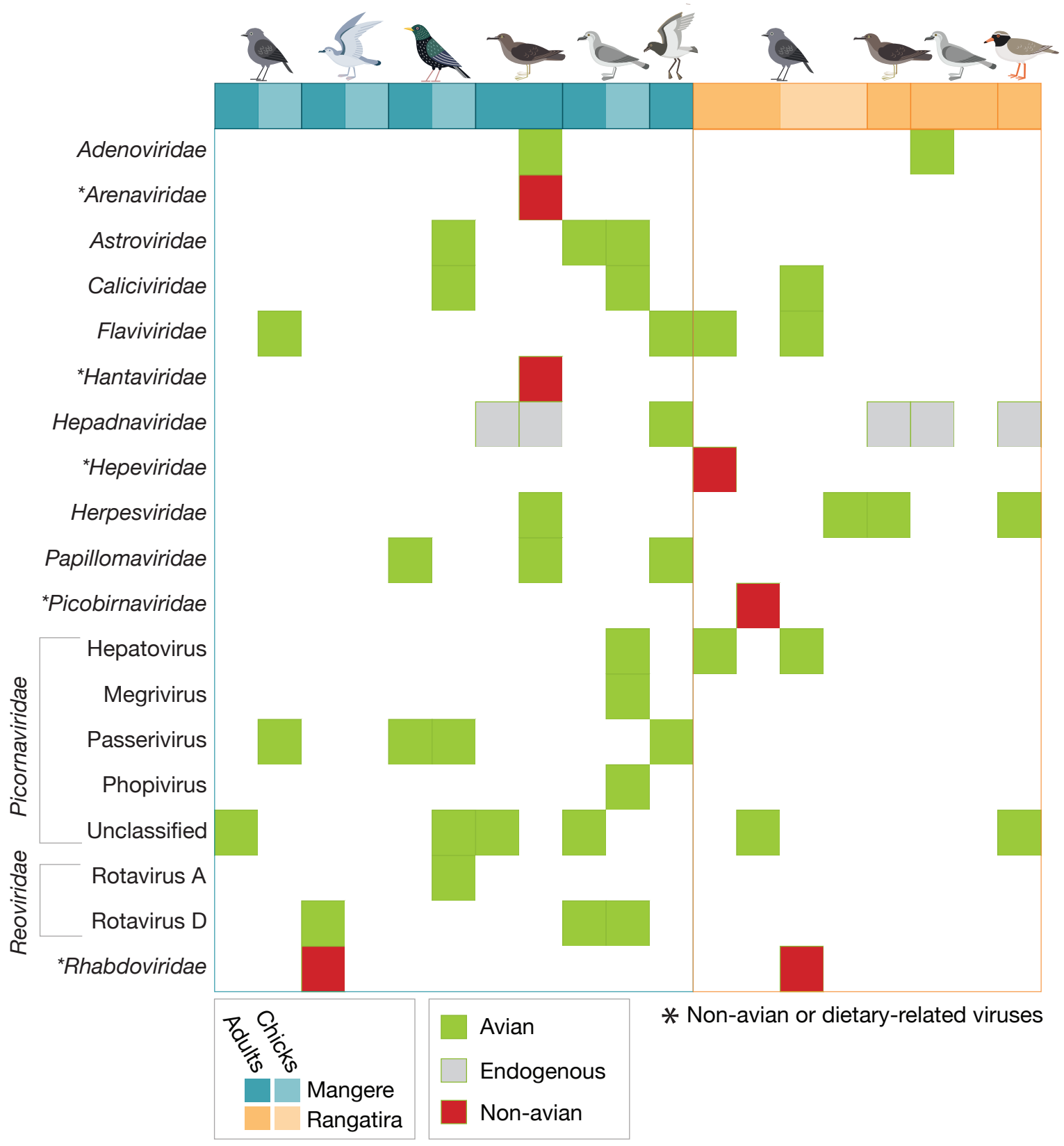

Supplement: Supplementary file 2 — Additional file 2: Supplementary Figure 2. Presence-absence plot of avian and avian-associated viral taxa by host species, location, and age group. Dark and light blue blocks indicate adult and chick viromes from Mangere Island (left), respectively; dark and light orange blocks indicate adult and chick viromes from Rangatira Island (right). Green blocks indicate the presence of avian viruses, while red blocks and viral taxa preceded by an asterisk (*) indicate avian-associated viruses, likely from dietary or environmental sources. Grey blocks indicate the presence of likely endogenous viral elements. [file 12862_2024_2277_MOESM2_ESM.pdf]

Arenaviridae

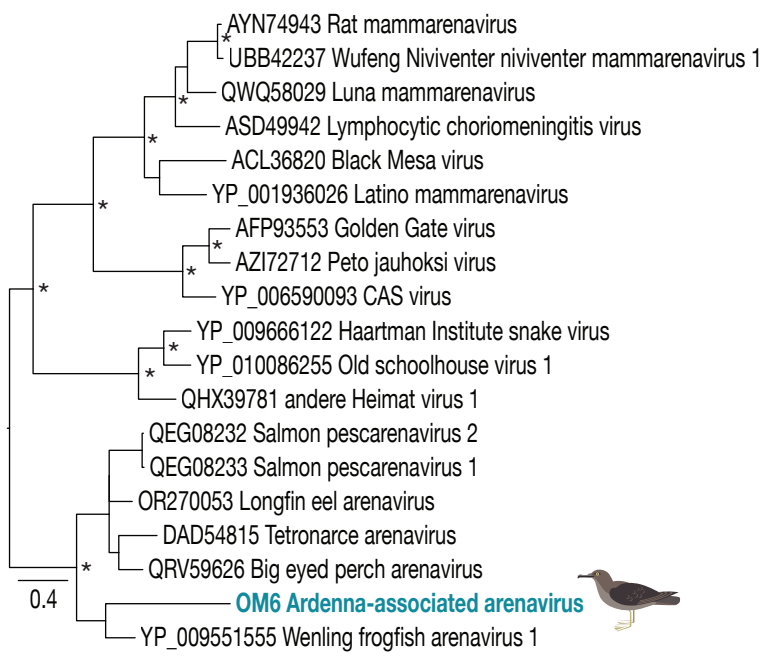

Hantaviridae

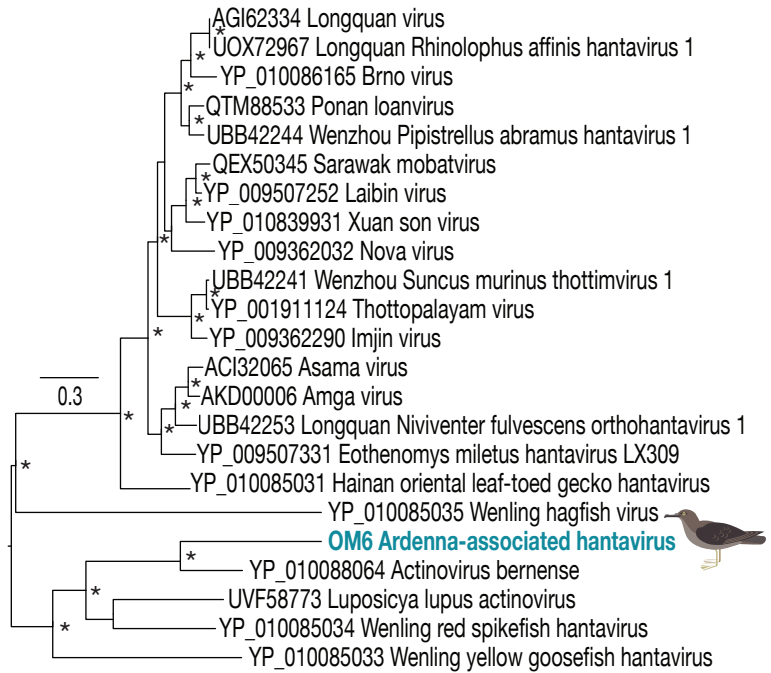

Hepeviridae

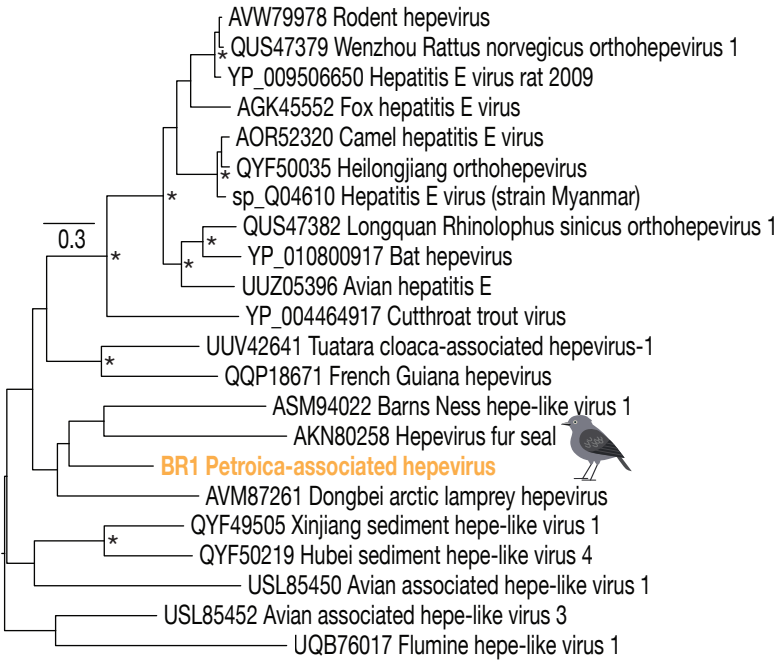

Picobirnaviridae

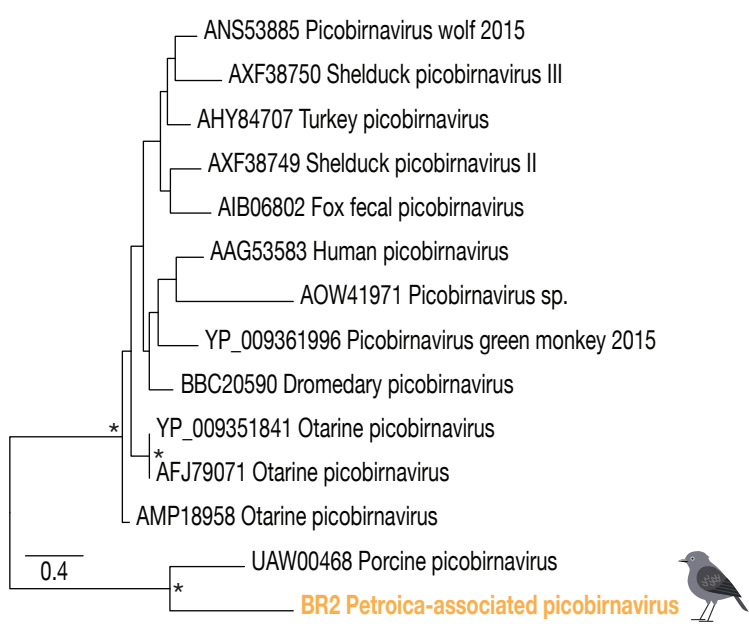

Supplement: Supplementary file 3 — Additional file 3: Supplementary Figure 3. Maximum likelihood trees of avian-associated viruses. Viruses identified as being associated with birds on Mangere Island are highlighted in blue and viruses identified on Rangatira Island are highlighted in orange. Host species associated with the viruses are indicated by bird illustrations. Substitutions per site indicated by the key on left-hand side of trees. Nodes with ≥ 95 UFbootstrap support values are denoted by an asterisk (*). [file 12862_2024_2277_MOESM3_ESM.pdf]

Microbial Richness

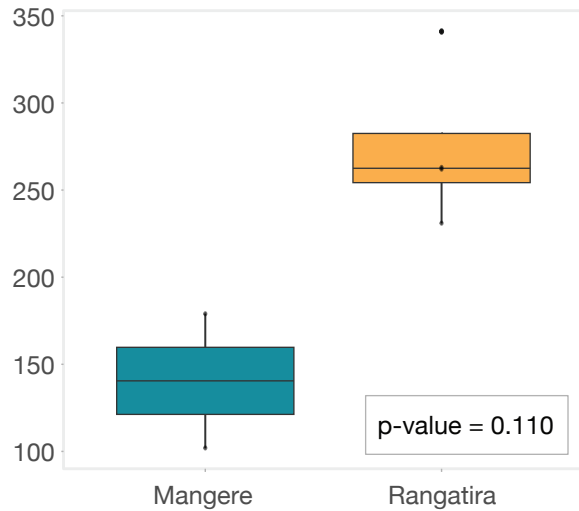

Shannon Index

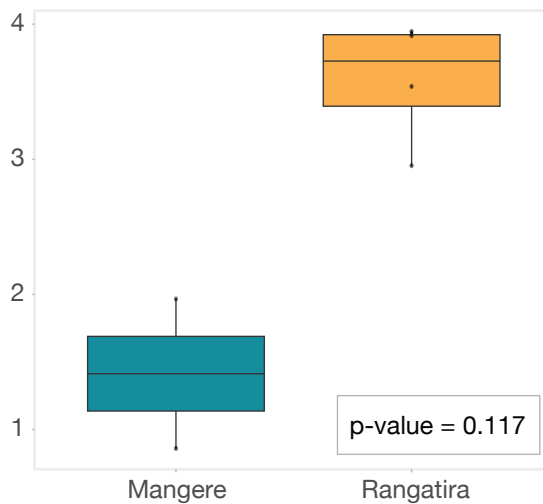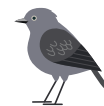

Mangere robins  
Rangatira robins

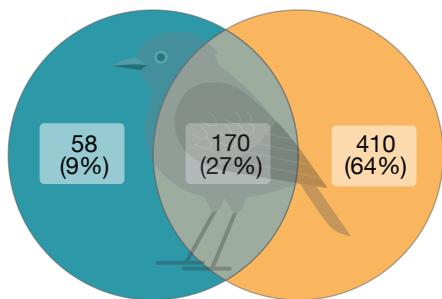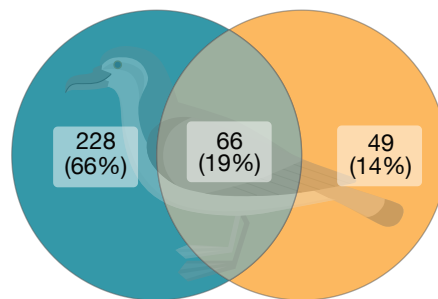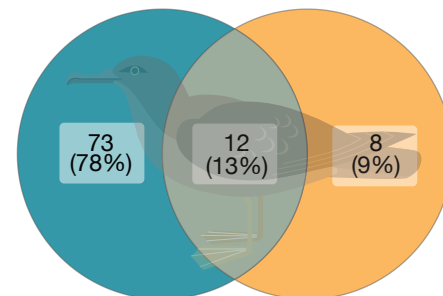

Supplement: Supplementary file 4 — Additional file 4: Supplementary Figure 4. Comparisons of non-viral microbial genera richness and diversity per host species and sampling location. Comparison of full genus-level microbiome microbial richness (left, t-test p-value 0.110) and Shannon indices (right, t-test p-value 0.117) of Mangere (blue) and Rangatira (orange) black robin (top). Venn diagrams of the unique microbial genera of black robin (left), broad-billed prions (middle), and sooty shearwater (right) based on their sampling location – Mangere (blue) or Rangatira (orange) (bottom). [file 12862_2024_2277_MOESM4_ESM.pdf]

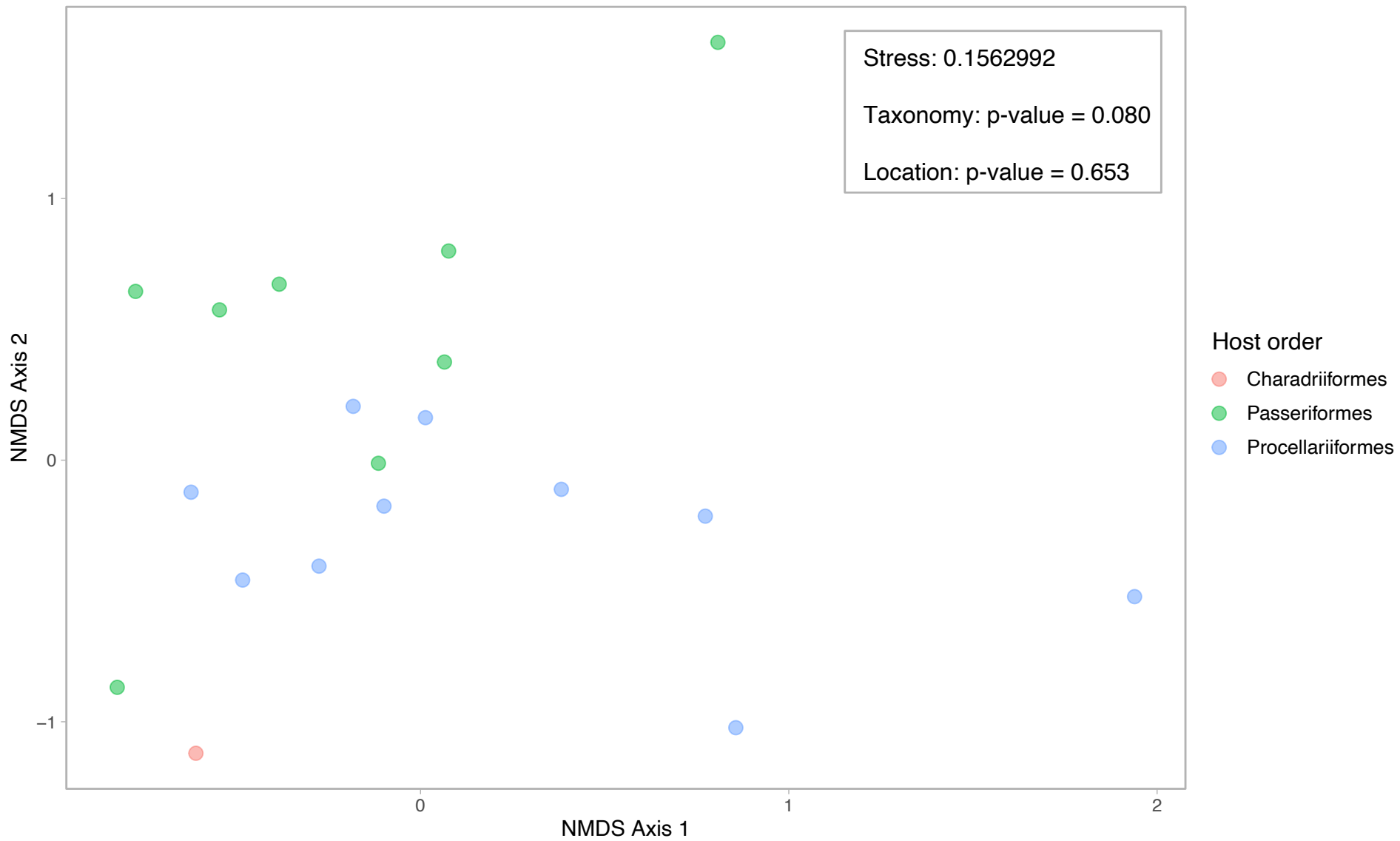

Supplement: Supplementary file 5 — Additional file 5: Supplementary Figure 5. Non-metric multidimensional scaling plots of non-vertebrate family-level virome compositions in Chatham birds. Non-vertebrate family-level virome compositions of sampling libraries (points) were plotted by Bray-Curtis distances and coloured by host order. Non-vertebrate family-level virome compositions were not significantly influenced by host taxonomy or location (PERMANOVA p-value = 0.08 and 0.65, respectively). [file 12862_2024_2277_MOESM5_ESM.pdf]
